# Economic Burden and Influencing Factors of Acute Gastroenteritis in China: A Population-Based Face to Face Survey in 2018

**DOI:** 10.3389/fpubh.2022.905458

**Published:** 2022-06-03

**Authors:** Yue Huo, Fei Gao, Jiayu Wang, Zhongwei Liu, Liangru Zhou, Baiyang Gu, Xin Zhang, Yi Ma

**Affiliations:** ^1^School of Health Management, Harbin Medical University, Harbin, China; ^2^Heilongjiang Provincial Center for Disease, Control and Prevention, Harbin, China

**Keywords:** acute gastroenteritis, economic burden, influential factors, self-reported suspected etiology, population surveillance

## Abstract

**Background:**

Acute gastroenteritis is an important and highly prevalent public health problem worldwide. The purpose of this study was to assess the economic burden of disease and its influencing factors in patients with acute gastroenteritis in Heilongjiang Province, China.

**Methods:**

A multi-stage stratified random sampling method was used in a face-to-face household survey in 2018. Demographic and socioeconomic characteristics, clinical symptoms, suspicious dietary history, and disease treatment information were collected from 19,647 respondents. One-way analysis of variance and multiple stepwise regression analysis were used to investigate the factors associated with the economic burden of acute gastroenteritis. Quantitative risk analysis and sensitivity analysis were performed to estimate the uncertainty and risk factors of the economic burden of acute gastroenteritis.

**Results:**

The total economic burden of patients with acute gastroenteritis was 63,969.22 CNY (Chinese Yuan), of which the direct economic burden accounted for 63.82%; the per capita economic burden was 131.35 CNY per month. Age, region, disease duration, and disease treatment were the main factors significantly associated with the economic burden of acute gastroenteritis (*P* < 0.05). The average economic burden of patients with acute gastroenteritis was approximately 571.84 CNY/person (95% CI: 227–1,459). Sensitivity analysis showed that the greatest impact was from the indirect economic burden.

**Conclusions:**

Acute gastroenteritis brings a substantial health burden to patients due to its high incidence. The economic burden of self-purchased drugs and the indirect economic burden of patients cannot be ignored. To better estimate the economic burden of acute gastroenteritis in China, further studies on the pathogen-specific economic burden of acute gastroenteritis are required.

## Introduction

Acute gastroenteritis is a globally important and common public health problem. The pathogenic factors of acute gastroenteritis include viruses, bacteria, parasites, toxins and metals. Acute gastroenteritis is most commonly seen in patients infected with at least one pathogen through food intake (1).

Although acute gastroenteritis is not fatal, the occurrence of the disease is extremely frequent, resulting in serious morbidity and economic burden to the population and health care system (2, 3). Globally, acute gastroenteritis causes approximately 89.5 million disability-adjusted life years lost and 1.45 million deaths each year (4). Acute gastroenteritis is one of the leading causes of disease in the United States, with approximately 179 million cases per year (5). A recent study in France estimated the incidence of acute gastroenteritis at an estimated 0.33 case/person-year, with over 21 million acute gastroenteritis events per year (6).

Acute gastroenteritis is also a serious public health problem in China, where a population surveillance survey of acute gastroenteritis was carried out in some provinces from 2010 to 2011 and its burden was preliminarily assessed (7). The survey data of six provinces showed that the weighted monthly prevalence rate of acute gastroenteritis was 4.2%, with an incidence rate of 0.56 times/person-year (8).

The economic burden of acute gastroenteritis studies carried out internationally are mostly based on the health care system (2, 9–11), while there are still few relevant studies based on population surveys (7). The real burden of acute gastroenteritis may be underreported and remains unclear. In order to better understand the occurrence and treatment of acute gastroenteritis, an implementation plan was carried out for special monitoring of the burden of acute gastroenteritis in Heilongjiang Province of China. The aim of this study was to analyze the economic burden of disease and its main influencing factors in patients with acute gastroenteritis in Heilongjiang Province in 2018, and to evaluate the socioeconomic impact of acute gastroenteritis, in order to provide a basis for the formulation of policies related to the prevention and control of acute gastroenteritis.

## Materials and Methods

### Data Collection

#### Study Setting

Three cities, Harbin, Mudanjiang and Heihe were selected as study sites according to the economic conditions, geographical location, population size and feasibility of each region. Two to three districts/counties were selected within each of the three cities, four to five townships/sub-districts were selected under districts/counties, and two neighborhood committees/administrative villages were selected from each township/sub-district.

#### Sampling Method of Survey Households

The relevant departments provided the corresponding neighborhood communities and administrative village with household registration base books, numbered the households in a certain order after excluding the households that could not be investigated such as unoccupied households, and formed a registration form in turn. For neighborhood committees, the sub-districts were sorted clockwise according to the straight line distance from the neighborhood committee office from near to far, and then by the door number, residential building number, unit, and number of households. For rural areas, the first row in the south of the village, the first household in the east of the village was numbered one, and then from east to west, from south to north. After numbering, the households were ordered from one to *n* (total number), and each household corresponded to a number. Using random number software, 132 households were selected from each neighborhood committee and administrative village in Heihe, and 192 households were selected in Harbin and Mudanjiang. The selected survey households were equally distributed for 12 months. For Heihe, 11 households were surveyed each month in each neighborhood committee/administrative village. For Harbin and Mudanjiang, 16 households were surveyed each month in each neighborhood committee/ administrative village. If the household were lost to follow-up, just skip to the next household on the registration form.

#### Survey Objects

The subjects of the survey were resident populations who had been living continuously for 6 months or more in neighborhood committees/administrative villages. In the selected households, at least one person was investigated in each household. In each survey household, the survey object was identified in each survey household according to the principle of the most recent birthday method, that is, individuals whose household was about to have a birthday (12). Survey objects were selected according to five age groups, including childhood, juvenile, youth, middle-aged, and elderly. A maximum of two people were selected for each age group, and a maximum of five people were selected for each household as the survey objects. At least 26 people are investigated in each neighborhood committee/administrative village in Harbin and Mudanjiang every month, and at least 18 people in each neighborhood committee/administrative village in Heihe are investigated every month.

### Definition of Acute Gastroenteritis

Cases of acute gastroenteritis were defined as individuals with ≥ 3 episodes of diarrhea or one episode of vomiting within 24 h, but did not include individuals with colorectal cancer, irritable bowel syndrome, Crohn's disease, ulcerative colitis, fibrosis, coeliac disease, or other chronic diseases with symptoms of diarrhea or vomiting; nor were individuals included whose symptoms were due to drug treatment, excessive alcohol consumption, or pregnancy (13, 14).

### Investigation Content and Method

The survey included five categories of questions: the respondent's gender, age, insurance participation, education level, residence, region, annual per capita income of the family, season of onset, and whether other members of the family were ill in the previous 4 weeks; clinical symptoms and signs such as vomiting, diarrhea, disease duration, and self-reported suspicious etiology; suspected dietary history such as food triggers and exposure places; and disease treatment and socioeconomic impact of the disease. A population-based household face-to-face survey of acute gastroenteritis was used. Investigators visited households from the 21st to the end of each month to investigate the incidence and related conditions of acute gastroenteritis in the previous 4 weeks. If the respondent was less than 12 years old, the parent or guardian answered the question on his/her behalf; If the respondent was between the ages of 12 and 16, based on the opinions of their parents or guardians, it was decided whether to answer the questions themselves. Others who do not have the ability to answer questions can also be assisted by others, but try to answer in person. If the selected household was unsuccessfully contacted for three times, it was counted as lost to follow-up.

### Calculation of Economic Burden

The economic burden of disease includes two parts: direct economic burden and indirect economic burden. Direct economic burden includes direct medical costs and direct non-medical costs, of which direct medical costs refers to self-purchased drug costs, outpatient diagnosis and treatment costs, and hospitalization costs during the treatment of patients. Direct non-medical costs refers to the purchase of drugs, and the transportation, accommodation, and other costs involved in the diagnosis and treatment of patients, and those costs borne by persons accompanying the patients to healthcare visits. The indirect economic burden is primarily due to the costs borne by patients and their attending family members who miss school or work due to illness. The calculation formula was:

Indirect economic burden = days of work or school lost by patients and caregivers × average wage of urban employees in Heilongjiang Province in 2018/365 (days).

The 2019 statistical yearbook data of the Statistics Bureau of Heilongjiang Province showed that the average salary of employed personnel in urban units was 60,780 CNY, which was converted according to the human capital method for indirect disease burden (15); the daily salary was 166.52 CNY (365 days).

### Statistical Analysis

The basic situation, clinical symptoms and signs, suspected dietary history, treatment, and socioeconomic impact of the disease on the cases were statistically described, and the economic burden on different age groups and by disease treatment was analyzed by subgroup. Log-transformed values of the economic burden of disease were used for the dependent variable; the independent variables included gender, age, medical insurance, education degree, residential property, region, annual per capita household income, season, disease duration, self-reported suspected etiology, and disease treatment, and main symptoms. Factors with *P* < 0.05 in one-way ANOVA were included in the model for multiple stepwise regression analysis. In order to avoid the problem of collinearity among influencing factors, the variance inflation factor was used for diagnosis (16). Multiple stepwise regression analysis was performed using the backward entry method with the exclusion criterion of α_out_ = 0.10 (17). Stata15.0 was used for data collation and analysis.

### Quantitative Risk Analysis and Sensitivity Analysis of the Economic Burden of Disease

The model was created in Microsoft® Excel and Risk 8 was used for risk quantification analysis of the economic burden of acute gastroenteritis disease. The average economic burden of acute gastroenteritis disease was modeled, with simulations changing parameter estimates, such as direct medical costs, direct non-medical costs, missed work days, and discount rates of acute gastroenteritis disease. The simulation was iterated 10,000 times to obtain a 95% confidence interval (18, 19), and sensitivity analysis was performed based on the Monte Carlo simulation method to investigate the effect of key parameters on the estimation of the economic burden of acute gastroenteritis. An inverse Gaussian distribution was used for direct medical costs and direct non-medical costs, and an exponential distribution was used for missed work days. The three distributions were obtained by fitting using Risk software through the existing collected data. The PERT distribution was used for the discount rate. The minimum, most likely, and maximum values of parameter distributions were obtained through a literature search.

## Results

### Basic Information

A total of 19,647 individuals were investigated in this study, including 10,045 males (51.13%) and 9,602 females (48.87%). The 45–64 age group surveyed 8,989 people (45.75%). The education level of 82,64 cases (42.06%) of the respondents was junior high school.

There were 696 patients (3.54%) with acute gastroenteritis, including 376 (54.02%) males and 320 (45.98%) females. There were 336 patients (48.28%) with acute gastroenteritis in the age group of 45–64 years. Of the patients, 93.68% (654/696) had health insurance. There were 604 (86.78%) patients with acute gastroenteritis whose disease duration was <3 days. The self-reported suspected etiology of acute gastroenteritis was 414 cases (59.48%) of eating contaminated food, three cases (0.43%) of person-to-person contact, and 255 cases (36.64%) of unknown origin. Among the patients with acute gastroenteritis, 61.93% (431/696) chose self-purchased drugs for treatment, 9.20% (64/696) patients visited a medical institution, of whom four (0.57%) were hospitalized. Ninety-six (13.79%) patients with acute gastroenteritis presented with vomiting symptoms, 680 (97.70%) with diarrhea symptoms, and only 2 (0.29%) with bloody stool complications (Table 1).

**Table 1:** Demographic and prevalence characteristics of acute gastroenteritis survey subjects in Heilongjiang Province.

**Characteristic**	**Respondents**	**Proportion (%)**	**Patient**	**Proportion (%)**
	***(N* = 19,647)**		***(N* = 696)**	
Gender				
Male	10,045	51.13	376	54.02
Female	9,602	48.87	320	45.98
Age (year)				
0–4	130	0.66	11	1.58
5–14	466	2.37	13	1.87
15–24	504	2.57	16	2.30
25–44	4,144	21.09	162	23.28
45–64	8,989	45.75	336	48.28
≥65	5,414	27.56	158	22.70
Education degree				
Primary and below	6,107	31.08	195	28.02
Junior high school	8,264	42.06	279	40.09
High school/Technical secondary school	3,417	17.39	140	20.11
Junior college or above	1,859	9.46	82	11.78
Residential property				
Urban	9,720	49.47	386	55.46
Rural	9,927	50.53	310	44.54
Region				
Harbin	6,777	34.49	254	36.49
Mudanjiang	6,341	32.27	185	26.58
Heihe	6,529	33.23	257	36.93
Annual per capita household income				
<5,000	4,202	21.39	116	16.67
5,000–	6,806	34.64	270	38.79
20,000–	6,114	31.12	221	31.75
≥35,000	2,525	12.85	89	12.79
Season				
Spring or Summer	9,753	49.64	435	62.50
Autumn or Winter	9,894	50.36	261	37.50
Insurance				
Yes			652	93.68
No			44	6.32
Disease duration (day)				
<3			604	86.78
3–			73	10.49
6–			12	1.72
≥9			7	1.01
Self-reported suspected etiology				
Contaminated food			414	59.48
People-to-people contact			3	0.43
Polluted water			21	3.02
Animal contact			3	0.43
Unknown origin			255	36.64
Disease treatment				
Self-purchased drug			431	61.93
Primary medical institution			55	7.90
Hospital			9	1.29
Untreated (missed work)			3	0.43
Untreated (no economic burden)			209	30.03
Vomiting				
Yes			96	13.79
No			600	86.21
Diarrhea				
Yes			680	97.70
No			16	2.30
Other symptoms during vomiting and diarrhea				
Yes			538	77.30
No			158	22.70
Complications (bloody stools)				
Yes			2	0.29
No			694	99.71

*Since some people both self-purchased drugs and visited a hospital, the total percentage of disease treatment types may exceed 100%. Other symptoms present during vomiting and diarrhea mainly include: nausea, abdominal pain and loss of appetite, headache, fever, muscle pain, arthralgia, and otitis*.

### Economic Burden of Disease for Patients

The total economic burden of the 487 patients with acute gastroenteritis was 63,969.22 CNY, including direct medical costs of 37,568.66 CNY, direct non-medical costs of 3,254 CNY, and indirect economic burden of 23,146.36 CNY. The per capita economic burden was 131.35 CNY per month. The average self-purchased drug cost was 34.89 CNY (431/487), the average outpatient cost was 117.06 CNY (64/487), and the average hospitalization cost was 3,760.25 CNY (4/487). The average direct non-medical cost was 30.99 CNY (105/487), and the average indirect economic burden was 399.08 CNY (58/487). See Table 2 for details. A total of 639 families were involved in patients with acute gastroenteritis, a total of 455 families had disease economic burden, and the average economic burden of the family was 140.59 CNY/month.

**Table 2:** Economic burden of disease and age distribution of acute gastroenteritis (CNY).

**Age group**	**N**	**Direct medical costs**			**Direct non-medical costs**				**Indirect economic burden**		**Total economic burden**	**per capita**
		**Self-purchased** **drug**	**outpatient**	**hospitalization**	**Drug**	**Patients**	**Visit**	**Escort**	**Missed** **work days**	**cost**		
0–4	9	881	700	2,600	25	250	0	406	6	999.12	5,861.12	651.24
5–14	10	750.40	79	0	28	0	0	0	0	0	857.40	85.74
15–24	11	520	130	0	2	15	0	0	0	0	667	60.64
25–44	104	2,333.90	1,786.38	0	93	289	0	0	43	7,160.38	11,662.66	112.14
45–64	236	7,451.38	3,311.80	7,451	632	190	40	655	57	9,491.67	29,222.85	123.83
≥65	117	3,099	1,485	4,990	313	156	160	0	33	5,495.18	15,698.18	134.17
**Total**	487	15,035.68	7,492.18	15,041	1,093	900	200	1,061	139	23,146.36	63,969.22	131.35

### Economic Burden of Disease by Age Group

Age was divided into six groups. The distribution of the economic burden of acute gastroenteritis in different age groups revealed that the largest total economic burden occurred for the age group of 45–64 years, the value of which was 29,222.85 CNY. The age group of 0–4 years had the highest per capita total economic burden of 651.24 CNY, whereas the age group of 15–24 years had the lowest per capita total economic burden of 60.64 CNY, as detailed in Table 2.

### Economic Burden of Disease by Treatment

The economic burden of acute gastroenteritis for each treatment condition showed that the total economic burden of self-purchased drugs was the largest, at 28,975.28 CNY. Of the total economic burden per capita, hospitals represented the heaviest burden (1,913.35 CNY), followed by primary medical institution (311.05 CNY), untreated (222.03 CNY), with self-purchased drugs representing the lightest burden (68.99 CNY), as shown in Table 3.

**Table 3:** Economic burden of acute gastroenteritis by treatment (CNY).

	**N**	**Direct medical costs**			**Direct non-medical costs**				**Indirect economic burden**		**Total economic burden**	**per capita**
		**Self-purchased drug**	**outpatient**	**hospitalization**	**Drug**	**Patients**	**Visit**	**Escort**	**Missed work days**	**cost**		
Self-purchased drug	420	14,763.68	0	0	1,033	20	150	0	78	12,988.60	28,975.28	68.99
Primary medical institution	55	272	6,073.90	3,760	60	594	40	0	38	6,327.78	17,107.68	311.05
Hospital	9	0	1,418.28	11,281	0	286	10	1,061	19	3,163.89	17,220.17	1,913.35
Untreated	3	0	0	0	0	0	0	0	4	666.08	666.08	222.03
**Total**	487	15,035.68	7,492.18	15,041	1093	900	200	1,061	139	23,146.36	63,969.22	131.35

### Univariate Analysis of Factors Influencing the Economic Burden of Acute Gastroenteritis

The results of one-way ANOVA revealed that age, region, season, disease duration, and disease treatment were significantly associated with the economic burden of acute gastroenteritis (*P* < 0.05). There were age differences in the economic burden of acute gastroenteritis: patients aged 0-4 years had the highest per capita burden of 651.24 CNY (*P* = 0.046). The economic burden of acute gastroenteritis was different in different regions (*P* < 0.001), the highest economic burden was 218.85 CNY in Harbin. The per capita economic burden of acute gastroenteritis was higher in spring or summer (156.07 CNY) than in autumn or winter (88.07 CNY) (*P* = 0.019). The economic burden of acute gastroenteritis increased with the increase of the duration (*P* < 0.001).The economic burden of acute gastroenteritis in hospital was much higher than that in other places (1913.35 CNY) (*P* < 0.001). There was no statistically significant difference in diarrhea (*P* = 0.3416) and vomiting (*P* = 0.3279) on the economic burden of diseases. Table 4 summarizes the results.

**Table 4:** One-way analysis of variance of economic burden of acute gastroenteritis in Heilongjiang Province.

**Characteristic variable**	**Economic burden of disease**			
	**Mean(95%CI)**	**Std. Error**	***F***	***P***
Overall	131.35 (81.72–180.99)	25.26		
Gender			3.39	0.066
Male	172.31 (82.03–262.59)	45.85		
Female	84.05 (60.21–107.89)	12.10		
Age			2.27	0.046
0–4	651.24 (−417.60–1720.07)	463.50		
5–14	85.74 (10.09–161.39)	33.44		
15–24	60.64 (13.24–108.03)	21.27		
25–44	112.14 (71.38–152.90)	20.55		
45–64	123.83 (37.16–210.49)	43.99		
≥65	134.17 (56.25–212.09)	39.34		
Insurance			2.93	0.088
Yes	177.24 (70.20–284.29)	51.62		
No	129.08 (77.22–180.94)	26.39		
Education degree			0.54	0.656
Primary and below	154.17 (73.04–235.30)	41.06		
Junior high school	104.34 (61.75–146.93)	21.59		
High school/Technical secondary school	166.73 (−54.90–388.26)	111.44		
Junior college or above	106.20 (60.30–152.10)	22.90		
Residential property			0.01	0.942
Urban	137.91 (52.83–222.99)	43.20		
Rural	124.32 (76.09–172.55)	24.48		
Region			11.16	<0.001
Harbin	218.85 (88.11–349.59)	66.23		
Mudanjiang	98.89 (41.70–156.09)	28.92		
Heihe	72.47 (46.10–98.83)	26.64		
Annual per capita household income			0.84	0.474
<5,000	112.28 (12.48–212.08)	50.14		
5,000–	131.98 (75.88–188.08)	28.46		
20,000–	86.68 (60.52–112.84)	13.23		
≥35,000	252.32 (−69.27–573.91)	160.77		
Season			5.54	0.019
Spring or Summer	156.07 (82.84–229.30)	37.22		
Autumn or Winter	88.07 (40.86–135.29)	23.92		
Disease duration (day)			11.39	<0.001
<3	73.46 (58.31–88.61)	7.71		
3–	415.97 (46.41–785.54)	184.69		
6–	588.21 (176.08–1000.33)	168.43		
≥9	746.64 (−656.74–2150.02)	545.94		
Self-reported suspected etiology			1.76	0.135
Contaminated food	130.32 (59.27–201.36)	36.10		
People-to-people contact	94.33 (−5.54–194.20)	23.21		
Polluted water	209.31 (13.21–405.41)	89.09		
Animal contact	409.55 (−1277.40–2,096.49)	392.07		
Unknown origin	123.42 (56.91–189.92)	33.69		
Disease treatment			49.42	<0.001
Self-purchased drug	68.99 (48.75–89.23)	10.30		
Primary medical institution	311.05 (161.62–406.48)	74.53		
Hospital	1,913.35 (−574.98–4401.69)	1,079.07		
Untreated (missed work)	222.03 (−16.80–460.85)	55.51		
Vomiting			0.91	0.3416
Yes	134.56 (76.30–193.63)	29.84		
No	112.98(63.94–162.01)	24.63		
Diarrhea			0.96	0.3279
Yes	58.76(6.16–111.36)	24.14		
No	133.34(82.37–184.32)	25.94		
Other symptoms during vomiting and diarrhea			3.54	0.0604
Yes	62.38(37.60–86.97)	12.36		
No	145.94(86.08–205.79)	30.45		

### Multiple Stepwise Regression Analysis of Factors Influencing Economic Burden of Acute Gastroenteritis

Variance inflation factor diagnostics revealed tolerance 0.1 for all five independent variables and variance inflation factors considerably <10, i.e., there was no collinearity between independent variables; therefore, all variables could be included in the model (20). Finally, a multivariate stepwise regression equation was obtained (*F* = 53.09, *P* < 0.001). Stepwise regression analysis showed that the economic burden of acute gastroenteritis was significantly associated (*P* < 0.01) with age, region, disease duration, and disease treatment. Patients aged ≥ 25 years had significantly lower economic burden of disease than did patients aged 0–4 years (25–44: β = −1.215, *P* = 0.005; 45–64: β = −1.271, *P* = 0.002; ≥65: β = −1.253, *P* = 0.003). Patients in provincial capital cities had significantly higher economic burden of disease than did those in non-provincial capital cities: Mudanjiang (β = −0.337, *P* = 0.031) and Heihe (β = −0.430, *P* = 0.004). Economic burden of disease is greatest with disease duration ≥ 9 (β = 2.327, *P* < 0.001). Patients with self-purchased drugs had the least economic burden of disease (Primary medical institution: β = 1.580, *P* < 0.001; Hospital: β = 2.480, *P* < 0.001; Untreated: β = 2.299, *P* = 0.001) (Table 5).

**Table 5:** Multiple stepwise regression analysis of economic burden of patients with acute gastroenteritis in Heilongjiang Province.

**Variable**	**Logarithm of Economic Burden of Disease**						
	**β**	**Std.Error**	**B**	***t***	***P***	**95%CI**	
Age(0–4 years as control group)							
5–14	−0.717	0.561	−0.070	−1.28	0.020	−1.820	0.386
15–24	−0.984	0.543	−0.101	−1.81	0.071	−2.052	0.083
25–44	−1.215	0.426	−0.344	−2.85	0.005	−2.053	−0.377
45–64	−1.271	0.418	−0.438	−3.04	0.002	−2.092	−0.450
≥65	−1.253	0.425	−0.369	−2.95	0.003	−2.087	−0.419
Region(Harbin as control group)							
Mudanjiang	−0.337	0.142	−0.104	−2.35	0.019	−0.612	−0.055
Heihe	−0.430	0.135	−0.143	−3.18	0.002	−0.695	−0.164
Disease duration (<3 as control group)							
3–	0.517	0.170	0.117	3.03	0.003	0.182	0.851
6–	2.204	0.464	0.181	4.75	0.000	1.292	3.116
≥9	2.327	0.498	0.177	4.67	0.000	1.348	3.305
Disease treatment(Self-purchased drug as control group)							
Primary medical institution	1.580	0.181	0.345	8.75	0.000	1.225	1.935
Hospital	2.480	0.423	0.230	5.87	0.000	1.649	3.310
Untreated (missed work)	2.299	0.704	0.124	3.27	0.001	0.916	3.682
_cons	4.539	0.420	–	10.81	0.000	3.714	5.364

### Summary and Sensitivity Analysis of Quantitative Risk Analysis Model

**Figure 1:**
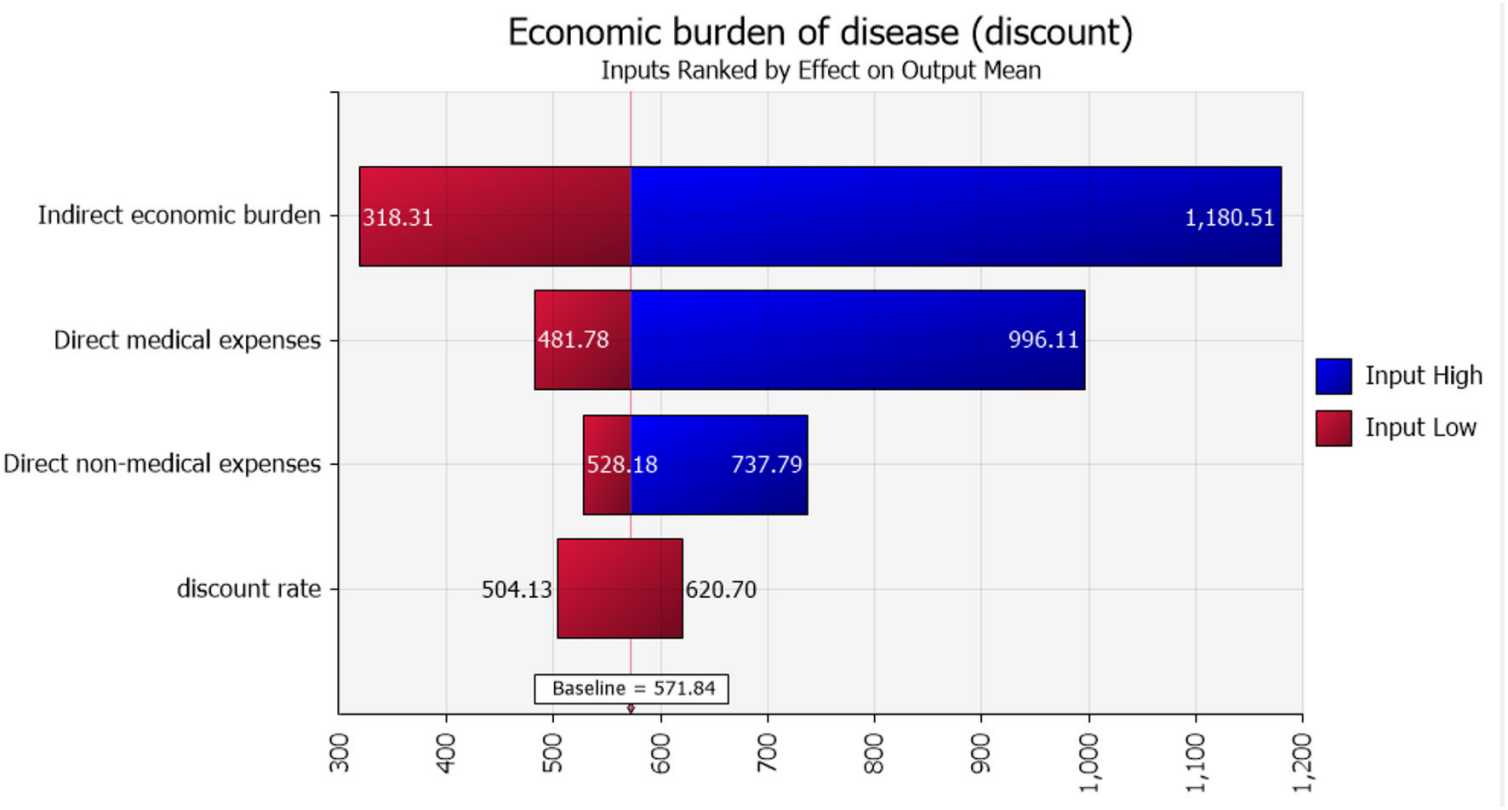
Sensitivity analysis of average acute gastroenteritis disease economic burden.

**Figure 2:**
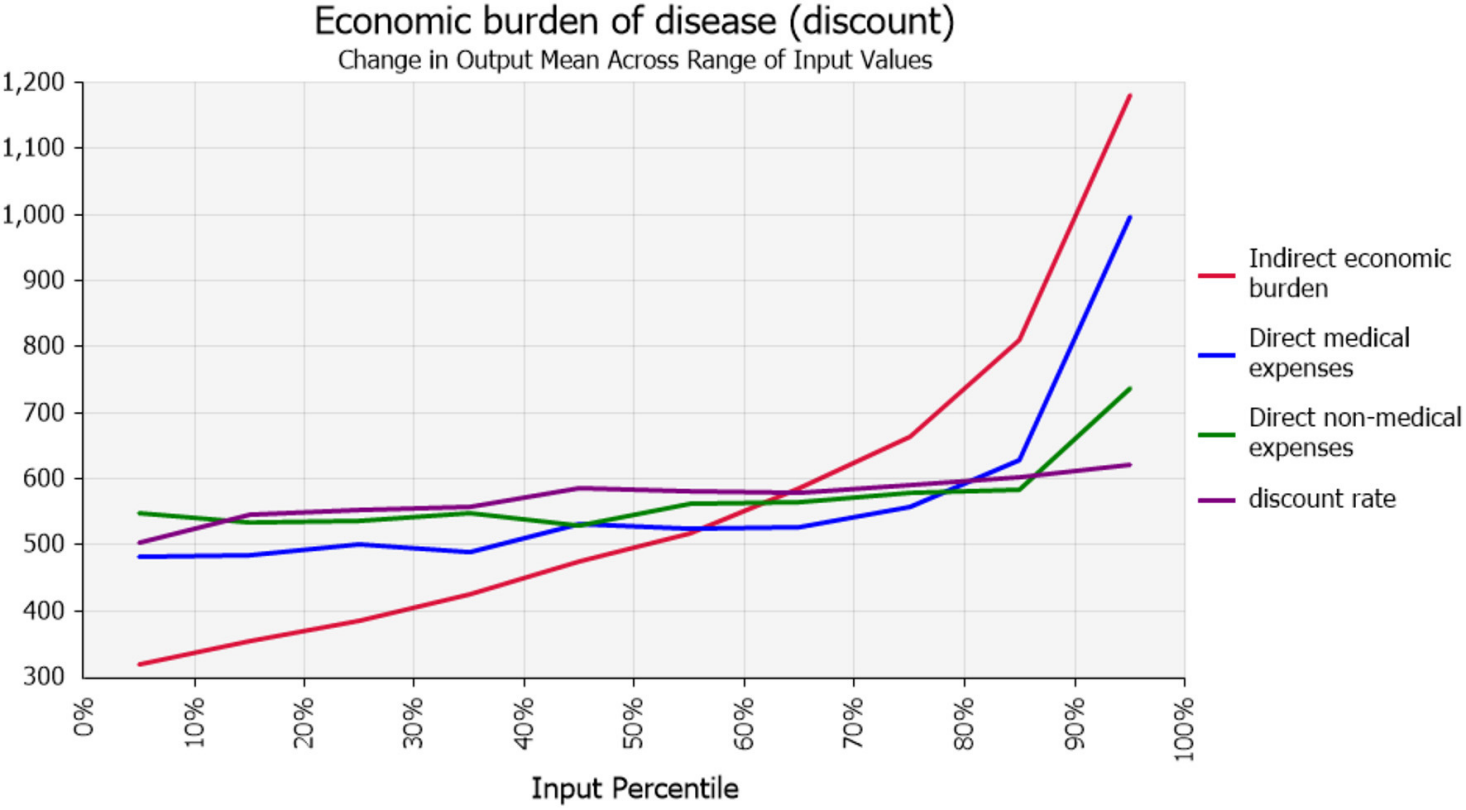
Impact of percentage distribution of model input parameters (10 groupings) on average economic burden of disease.

The results of quantitative risk analysis, or risk metrics, showed that the average economic burden of acute gastroenteritis in Heilongjiang Province was approximately 571.84 CNY/person (95% CI: 227–1,459). As can be seen from the results of the sensitivity analysis, the greatest influence on the average economic burden of the disease was the indirect economic burden, as detailed in Tornado Figure 1. Spider Figure 2 shows the effect of the percentage distribution of model input parameters (10 groups) on the average economic burden of the disease. There was a change node at approximately the 60th percentile, such that the effect of direct economic burden was higher than indirect economic burden in the first 60th percentile; thereafter, the opposite was true.

## Discussion

The study results showed that the monthly prevalence of the surveyed population was 3.54%. The medical institution visit rate of 696 patients with acute gastroenteritis disease was 9.20%, and the per capita economic burden of patients was 131.35 CNY per month; it was lower than the rate of visits for patients with acute gastroenteritis in France listed by Cauteren et al. (21) and also lower than the rate of visits for patients with acute gastroenteritis and the per capita total economic burden reported by Ma et al. (22) in Beijing, China.

The proportion of individuals who sought consultations for acute gastroenteritis in Heilongjiang Province was relatively low; 61.93% of patients were more likely to choose self-purchased drugs and home-prepared drugs for treatment, and most visits were concentrated in primary medical institutions. This is likely related to the attention patients pay to common diseases that are initially mild and often self-limiting, such as acute gastroenteritis. However, the condition may progress to severe acute gastroenteritis for which patients may fail to receive timely and standardized treatment. Subsequent deterioration of the patient's condition and recurrent disease may also increase the economic burden of the disease. Compared with Beijing, the lower economic burden of acute gastroenteritis in Heilongjiang Province may be related to the regional economic status (including low outpatient treatment costs and salary levels) and lower visit rates.

Most patients with acute gastroenteritis chose to self-medicate, and the proportion who visited healthcare professionals was low. The diagnosis and treatment costs of patients may not be collected and recorded, which represents a lack of data by which to monitor medical institutions (23, 24). In this study, a door-to-door survey can more effectively carry out acute gastroenteritis disease data monitoring, compared with medical institution survey. This survey approach can avoid the underestimation of the economic burden caused by patients who do not utilize healthcare systems.

The economic burden of acute gastroenteritis disease was associated with factors such as age, region, season, days of illness duration, and disease treatment. The economic burden of total disease per capita was highest in those aged 0–4 years, which is consistent with prior research (25–28). The main reasons may be that children's gastrointestinal function and immune system are not yet well-developed, have lower resistance, have more specific pathogens, and children have poor hygiene habits such as sucking fingers, which are likely to increase the risk of their pathogen exposure. There was a significant difference in the economic burden of disease among the three sample cities in this survey, and the economic level between regions was positively correlated with the monthly economic burden of disease per patient. This illustrates that the better the economic status and development level of a region, the greater the use of health services, namely people will consider their own health status and utilize medical services as needed. The economic burden is higher in spring and summer than in autumn and winter, which is primarily due to the effects of meteorological factors on the growth and reproduction of pathogens (29). For clinical symptoms, the longer the duration of the disease, the greater the duration of the economic burden, consistent with the results of Liu (30) and Lin (31), for example. Disease duration generally reflects the priority of the disease; the longer the duration of the disease, the greater the hospitalization costs and self-purchased drug costs, and economic losses caused by missed work.

According to the analysis of disease treatment, the patients who selected self-purchased drug treatment had the highest economic burden (45.30%), but their per capita economic burden was the least. For patients, the frequency of utilizing primary medical institutions was much higher than that of hospitals, but the economic burden of the former was less than one-half (49.84%) of the total economic burden of visits, while the economic burden associated with hospital use was higher. This may be related to those who visit hospitals receiving more regular examinations and treatment by highly qualified physicians.

As current studies are generally deterministic studies, the effect of risk factors on the results was not considered. In this study, the range of economic burden of disease was obtained by quantitative risk analysis, namely, the possibility of the average economic burden of acute gastroenteritis exceeding 227 CNY/person was 95%, and the possibility of the burden exceeding 1,459 CNY/person was 5%. Through the results of sensitivity analysis, among the average economic burden of diseases, the indirect economic burden accounts for a greater proportion, indicating that the impact caused by indirect costs is increasing. In addition, it is difficult to quantify the intangible costs caused by mental burdens such as sadness, anxiety, and inconvenience to patients and those who accompany them on healthcare visits. These intangible burdens may also affect the total costs; this needs to be further investigated and explored in future studies.

In order to reduce the economic burden of acute gastroenteritis disease, on the one hand, it is necessary to intensify the supervision of food safety, while strengthening the publicity and education of personal hygiene and food safety. Focus on young children and elderly population to reduce the incidence of acute gastroenteritis is key. On the other hand, the diagnosis and treatment ability of primary medical institutions for the treatment of acute gastroenteritis should be improved to reduce the economic burden caused by the occurrence of the disease.

This study has some limitations: First, a retrospective survey was used to inquire about the prevalence of acute gastroenteritis in the previous 4 weeks, which may have generated recall bias. In order to reduce bias, investigators try to ask the actual onset date, so as to obtain more accurate results. Second, the suspected etiology of this study was derived from patient self-assessment with great uncertainty. Patients are rarely able to really know the cause of acute gastroenteritis and are often thought to be caused by food. Only two patients in the survey answered that they had been asked to provide stool samples for pathogen testing, so the foodborne exposure and person-to-person transmission ratio of acute gastroenteritis could not be clarified by pathogen test results.

## Conclusions

Acute gastroenteritis brings great health burden to patients due to its high incidence, but most patients with acute gastroenteritis choose self-purchased drugs. Due to its low rate of medical institution visits, the economic burden may be seriously underestimated, and population surveys can better avoid underestimation. The indirect economic burden caused by disease is also a non-negligible part. To better estimate the economic burden of acute gastroenteritis in China, further studies are needed to investigate the pathogen-specific economic burden of acute gastroenteritis.

## Data Availability Statement

The raw data supporting the conclusions of this article will be made available by the authors, without undue reservation.

## Ethics Statement

The studies involving human participants were reviewed and approved by Heilongjiang Provincial Center for Disease Control and Prevention, China. Written informed consent to participate in this study was provided by the participants' legal guardian/next of kin.

## Author Contributions

YH and XZ made substantial contributions to the design of the work. YH and FG performed design, analysis and interpretation of data, drafting the first version of the manuscript. ZL, JW, BG, and LZ were involved in the coordination of the project, and contributed to drafting subsequent versions of the manuscript. XZ and YM substantively revised it. All authors contributed to the article and approved the submitted version.

## Funding

This work was supported by National Key R&D Program of China (2019YFC1605201) and Scientific Research Project of Heilongjiang Provincial Health and Family Planning Commission (2017-531).

## Conflict of Interest

The authors declare that the research was conducted in the absence of any commercial or financial relationships that could be construed as a potential conflict of interest.

## Publisher's Note

All claims expressed in this article are solely those of the authors and do not necessarily represent those of their affiliated organizations, or those of the publisher, the editors and the reviewers. Any product that may be evaluated in this article, or claim that may be made by its manufacturer, is not guaranteed or endorsed by the publisher.

## References

[1] Hu P Liu C Ruan J Yuan M Ju C Ma Y. FilmArray GI-panel performance for the rapid and multiple detection of gastrointestinal microorganisms in foodborne illness outbreaks in Shenzhen during 2018-2019. Infect Genet Evol. (2020) 86:104607. 10.1016/j.meegid.2020.104607 33132110

[2] Melliez H Boelle P-Y Baron S Mouton Y Yazdanpanah Y. Morbidity and cost of rotavirus infections in France. Médecine Mal Infect. (2005) 35:492–9. 10.1016/j.medmal.2005.08.007 16316731

[3] van den Brandhof WE De Wit GA de Wit MA van Duynhoven YT. Costs of gastroenteritis in The Netherlands. Epidemiol Infect. (2004) 132:211–21. 10.1017/S0950268803001559 15061495 PMC2870096

[4] Hay SI Abajobir AA Abate KH Abbafati C Abbas KM Abd-Allah F. Global, regional, and national Disability-Adjusted Life-Years (DALYs) for 333 diseases and injuries and Healthy Life Expectancy (HALE) for 195 countries and territories, 1990-2016: a systematic analysis for the Global Burden of Disease Study 2016. Lancet. (2017) 90:1260–344. 10.1016/S0140-6736(17)32130-X 28919118 PMC5605707

[5] Scallan E Griffin PM Angulo FJ Tauxe RV Hoekstra RM. Foodborne illness acquired in the United States-unspecified agents. Emerg Infect Dis. (2011) 17:16–22. 10.3201/eid1701.P21101 21192849 PMC3204615

[6] Jones TF McMillian MB Scallan E Frenzen PD Cronquist AB Thomas S. population-based estimate of the substantial burden of diarrhoeal disease in the United States; FoodNet, 1996–2003. Epidemiol Infect. (2007) 135:293–301. 10.1017/S0950268806006765 17291364 PMC2870567

[7] Zhou YJ Dai Y Yuan BJ Zhen SQ Tang Z Wu GL Wang Y. Population-based estimate of the burden of acute gastrointestinal illness in Jiangsu province, China, 2010-2011. Epidemiol Infect. (2013) 141:944–52. 10.1017/S0950268812001331 22793156 PMC9151840

[8] Zhao G H Sun C W. Analysis of food safety problems caused by foodborne diseases. Trans Innov Sci Technol. (2013) 35:35.

[9] Karve S Krishnarajah G Korsnes JS Cassidy A Candrilli SD. Burden of acute gastroenteritis, norovirus and rotavirus in a managed care population. Hum Vaccin Immunother. (2014) 10:1544–56. 10.4161/hv.28704 24732307 PMC5396247

[10] Papadopoulos T Klamer S Jacquinet S Catry B Litzroth A Mortgat L. The health and economic impact of acute gastroenteritis in Belgium, 2010-2014. Epidemiol Infect. (2019) 147:e146. 10.1017/S095026881900044X 30869061 PMC6518509

[11] Barker SF Zomer E O'Toole J Sinclair M Gibney K Liew D. Cost of gastroenteritis in Australia: a healthcare perspective. PLoS ONE. (2018) 13:e0195759. 10.1371/journal.pone.0195759 29649285 PMC5896984

[12] Lin Y Sun Y M He H T Fang H Q. Burden assessment of acute gastroenteritis among community population in Jiaxing from 2018 to 2019. J Food Safe. (2021) 33:6. 10.13590/j.cjfh.2021.04.013

[13] Cho SR Yun SJ Chae SJ Jung S Kim JH Yong KC. An outbreak associated with Sapovirus GI3 in an elementary school in Gyeonggi-do, Korea. J Korean Med Sci. (2020) 35:e281. 10.3346/jkms.2020.35.e281 32864904 PMC7458851

[14] Majowicz SE Hall G Scallan E Adak GK Gauci C Jones TF. A common, symptom-based case definition for gastroenteritis. Epidemiol Infect. (2008) 136:886–94. 10.1017/S0950268807009375 17686196 PMC2870876

[15] Jo C. Cost-of-illness studies: concepts, scopes, and methods. Clin Mol Hepatol. (2014) 20:327–37. 10.3350/cmh.2014.20.4.327 25548737 PMC4278062

[16] Senaviratna N Cooray T. Diagnosing multicollinearity of logistic regression model. Asian J Probab. (2019) 5:1–9. 10.9734/ajpas/2019/v5i230132

[17] Wang Y Gan Y Zhang J Mei J Feng J Lu Z. Analysis of the current status and associated factors of tuberculosis knowledge, attitudes, and practices among elderly people in Shenzhen: a cross-sectional study. BMC Public Health. (2021) 21:1513. 10.1186/s12889-021-11240-7 34353310 PMC8340377

[18] Zhang L Ai Y Liu J Yue N Xuan J Bal V. Economic burden of needlestick injuries among healthcare workers in China. J Med Econ. (2020) 23:683–9. 10.1080/13696998.2020.1737534 32122187

[19] Undurraga EA Betancourt-Cravioto M Ramos-Castañeda J Martínez-Vega R Méndez-Galván J Gubler DJ. Economic and disease burden of dengue in Mexico. PLoS Negl Trop Dis. (2015) 9:e0003547. 10.1371/journal.pntd.0003547 25786225 PMC4364886

[20] Ranjith CP Puzhakkal N Arunkrishnan MP Vysakh R Irfad MP Vijayagopal KS. Mean parotid dose prediction model using machine learning regression method for intensity-modulated radiotherapy in head and neck cancer. Med Dosim. (2021) 46:283–8. 10.1016/j.meddos.2021.02.003 33744079

[21] Cauteren DV Valk HD Vaux S Le Strat Y Vaillant V. Burden of acute gastroenteritis and healthcare-seeking behaviour in France: a population-based study. Epidemiol Infect. (2012) 140:697–705. 10.1017/S0950268811000999 21676346

[22] Ma XC Niu YL Wu YB Wang C Wang T Jiang J. Preliminary estimating the burden of foodborne acute gastrointestinal illness in Beijing City. J hygiene research. (2019) 48:589–93. 10.19813/j.cnki.weishengyanjiu.2019.04.008 31601340

[23] Majowicz SE Edge VL Fazil A McNab WB Doré KA Sockett PN. Estimating the under-reporting rate for infectious gastrointestinal illness in Ontario. Can J Public Health. (2005) 96:178–81. 10.1007/BF03403685 15913079 PMC6975884

[24] Wheeler JG Sethi D Cowden JM Wall PG Rodrigues LC Tompkins DS. Study of infectious intestinal disease in England: rates in the community, presenting to general practice, and reported to national surveillance. BMJ. (1999) 318:1046–50. 10.1136/bmj.318.7190.1046 10205103 PMC27838

[25] GBD 2016 Diarrhoeal Disease Collaborators. Estimates of the global, regional, and national morbidity, mortality, and aetiologies of diarrhoea in 195 countries: a systematic analysis for the Global Burden of Disease Study 2016. Lancet Infect Dis. (2018) 18:1211–28. 10.1016/S1473-3099(18)30362-1 30243583 PMC6202444

[26] Wang P Goggins WB Chan EYY. Associations of Salmonella hospitalizations with ambient temperature, humidity and rainfall in Hong Kong. Environ Int. (2018) 120:223–30. 10.1016/j.envint.2018.08.014 30103121

[27] Chen Y Yan WX Zhou YJ Zhen SQ Zhang RH Chen J. Burden of self-reported acute gastrointestinal illness in China: a population-based survey. BMC Public Health. (2013) 13:456–456. 10.1186/1471-2458-13-456 23656835 PMC3655923

[28] Scavia G Baldinelli F Busani L Caprioli A. The burden of self-reported acute gastrointestinal illness in Italy: a retrospective survey, 2008-2009. Epidemiol Infect. (2012) 140:1193–206. 10.1017/S0950268811002020 22014077 PMC3365479

[29] IngramMSt JohnJApplewhaiteTGaskinPSpringerKIndarL. Population-based estimates of acute gastrointestinal and foodborne illness in Barbados: a retrospective cross-sectional study. J Health Popul Nutr. (2013) 31:81–97.24992814

[30] Liu L Bai GD Xing Y Sun PH Zhai QQ Zhang D. Assessment of hospital visiting situation and disease burden of patients with foodborne diseases in Jilin Province. J Jilin Univ (Med ed). (2015) 41:207–12. 10.13481/j.1671-587x.20150241

[31] Lin M Dong B Q Liang D B. Multi-factor analysis of the disease burden and its risk factors among diarrhea patients in Guangxi. Prev Med. (2009):3806–8.

